# Designing Biodegradable and Active Multilayer System by Assembling an Electrospun Polycaprolactone Mat Containing Quercetin and Nanocellulose between Polylactic Acid Films

**DOI:** 10.3390/polym13081288

**Published:** 2021-04-15

**Authors:** Carol López de Dicastillo, Luan Garrido, Eliezer Velásquez, Adrián Rojas, Rafael Gavara

**Affiliations:** 1 Packaging Innovation Center (LABEN-Chile), University of Santiago of Chile (USACH), Obispo Umaña 050, Santiago 9170201, Chile; luan.garrido@usach.cl (L.G.); eliezer.velasquez@usach.cl (E.V.); adrian.rojass@usach.cl (A.R.); 2 Center for the Development of Nanoscience and Nanotechnology (CEDENNA), University of Santiago of Chile (USACH), Obispo Umaña 050, Santiago 9170201, Chile; 3 Packaging Laboratory, Institute of Agrochemistry and Food Technology, IATA-CSIC, Av. Agustín Escardino 7, 46980 Paterna, Spain; rgavara@iata.csic.es

**Keywords:** multilayer, biodegradable, polylactic acid, polycaprolactone, electrospinning

## Abstract

The design of multilayer systems is an innovative strategy to improve physical properties of biodegradable polymers and introduce functionality to the materials through the incorporation of an active compound into some of these layers. In this work, a trilayer film based on a sandwich of electrospun polycaprolactone (PCL) fibers (PCLé) containing quercetin (Q) and cellulose nanocrystals (CNC) between extruded polylactic acid (PLA) films was designed with the purpose of improving thermal and barrier properties and affording antioxidant activity to packaged foods. PCLé was successfully electrospun onto 70 µm-thick extruded PLA film followed by the assembling of a third 25 µm-thick commercial PLA film through hot pressing. Optical, morphological, thermal, and barrier properties were evaluated in order to study the effect of PCL layer and the addition of Q and CNC. Bilayer systems obtained after the electrospinning process of PCL onto PLA film were also evaluated. The release of quercetin from bi- and trilayer films to food simulants was also analyzed. Results evidenced that thermal treatment during thermo-compression melted PCL polymer and resulted in trilayer systems with barrier properties similar to single PLA film. Quercetin release from bi- and trilayer films followed a similar profile, but achieved highest value through the addition of CNC.

## 1. Introduction

Nowadays, concerns from a waste management point of view due to the substantial increase in the use of plastics has resulted in a strong research interest in the development of biodegradable materials as a good strategy to minimize the environmental impact of petroleum-based materials, principally used for food packaging applications [1,2]. There are several biodegradable polymers, such as polysaccharides, lipids, proteins, and others. Undoubtedly, PLA has become the most interesting material because it is commercially available, compostable, and produced at large industrial scale and can be processed by conventional melting extrusion processing [3,4]. However, PLA has certain disadvantages as its low thermal and barrier properties. The high permeability to gases and water vapor directly affects the packaged product by reducing the food shelf life. Therefore, the improvement of these properties through innovative technologies is a significant challenge for material scientists [5,6,7]. 

The development of multilayer materials has been considered a mechanism to improve material properties. Specifically, the improvement of barrier properties can occur by increasing the route of molecules diffusion through the layers of the materials. The mechanism is based on the synergistic effect between the properties of each of the materials that make up the complex structure [8]. Multilayer structures can be made by combining different polymers and using various techniques such as lamination, co-extrusion, coating, layer by layer, and more recently, the electrospinning technique [9,10,11,12]. Electrospinning is a simple, efficient, cost-effective and scalable technology capable of producing fibers. The fibrillary structures are obtained when a voltage is applied to a polymeric solution obtaining a jet with a conical structure known as “Taylor’s Cone”, from which the solvent evaporates to result in ultrafine structures with a high surface/volume ratio [13,14,15,16]. 

Nanotechnology has also shown up as an innovative technology to improve the mechanical and barrier properties of polymers over the last years. The attention has been focused on the incorporation of nanoreinforcements to polymeric matrices, such as nanoclays and nanocellulose [17,18]. Specifically, cellulose nanocrystals (CNC) have been one of the most researched nanofillers due to their excellent characteristics, such as highly crystalline structure, low density, biodegradability, transparency, and high specific area [5,6,7,9]. The polymeric matrix surrounds the reinforcing particles, resulting in composites with specific chemical or physical properties. This can occur through the formation of layers that can incorporate nanostructures to improve physical properties and also active agents to be released into food, both with the purpose of extending food shelf life. The development of active packaging by affording functionality to polymeric materials is a mechanism to generate added value and extend the shelf life of packaged food [19,20]. Because oxidation is one of the principal food deterioration processes, the design of materials incorporating antioxidants agents to be released into food is highly valuable [21,22].

Some biodegradable multilayer structures have been recently reported. Fabra et al. (2016) have developed a packaging material with very good oxygen and water vapor barrier properties, composed of electrospun polyhydroxybutyrate (PHB) fibers containing CNC, and a nanocomposite based on corn starch and CNC, as substrate polymer [23]. Wan et al. (2016) have applied a thermal compression system to develop a multilayer film based on electrospun zein fibers onto a bacterial cellulose film as a support [24]. Bilayer systems based on electrospun fibers of PHB and PLA blends containing catechin onto poly(3-hydroxybutyrate-co-3-hydroxyvalerate) compressed molded film have also been developed and presented antioxidant activity in a fatty food simulant [25]. 

The novelty of this present work is the combination of electrospinning and hot-pressing techniques using polycaprolactone electrospun fibers as a vehicle to incorporate the antioxidant compound and nanocellulose. A trilayer system based on electrospun fibers of polycaprolactone (PCL) containing quercetin (Q), as a powerful antioxidant agent, and CNC between extruded PLA layers has been developed in order to improve the water vapor barrier and provide some protection to food against oxidative processes. The use of electrospinning for the development of active biodegradable packaging materials with a multilayer structure is complex, because, in most cases, there is no good adhesion between the electrospun layer and the substrate. In order to solve this problem, PCL fiber containing the active agent was assembled between two PLA layers. First, PCL fiber was electrospun on a PLA substrate, and then, a third PLA layer was incorporated onto PCL fibers through hot pressing process. This system was done to guarantee the fixation of the fibers into the active multilayer system and tune the active compound release. Quercetin, whose antioxidant, anti-inflammatory, anti-carcinogenic, and antiviral properties have been extensively demonstrated, is a phenolic compound found in fruits and vegetables. Its high antioxidant action through the free-radical scavenging activity has propitiated its use in the development of active packaging [26,27,28,29,30]. Meanwhile, PCL is a synthetic biodegradable semicrystalline polyester with low melting point (approximately 60 °C) and glass transition temperature (approximately −60 °C); its mechanical and barrier properties make it an interesting polymer to be added in multilayer systems [31,32,33]. 

## 2. Materials and Methods

### 2.1. Materials

Polylactic acid (PLA), 2003D (specific gravity ¼ 1.24; MFR g/10 min (210 °C, 2.16 kg)), was purchased in pellet form from NatureWorks^®^ Co., Minnetonka (Minnesota, MN, USA). Polycaprolactone (PCL) ($\bar{M_{n}}\;\;$ = 80.000) and quercetin (Q) (≥99.5%). Cellulose nanocrystals (CNC) were purchased from the University of Maine. Chloroform (CHF), ethanol, and *N*,*N*-dimethylformamide (DMF) were supplied by Merck (Santiago, Chile). Moreover, 25 µm PLA films (PLA’) were purchased by Q4 Packaging Systems, S.L. (Valencia, Spain).

### 2.2. Preparation of Quercetin-Containing Multilayer Systems

First, PLA films were flat melt-extruded in a twin-screw extruder Scientific Lab Tech LTE20 (Bangkok, Thailand) with a temperature profile of 200–210 °C, a screw speed of 20 rpm, and 40% of torque. The resulting films presented an average thickness of around 65–70 µm measured by a digital micrometer Mitutoyo ID-C112.

Subsequently, bilayer films were prepared by coating one side of extruded PLA films with an electrospun PCL (PCLé) layer. Electrospinning was carried out using an electrospinning equipment (Spraybase^®^ power Supply Unit, Maynooth, Ireland) with a horizontal configuration linked to a rotating collector connected to a high-voltage power and covered with mentioned PLA film. A PCL solution was first prepared by dissolving 14% (*w*/*v*) of this biopolymer in a DMF/CHF 3:7 (*v*/*v*) mixture at room temperature. Q was subsequently added to the solution at 5%wt based on PCL weight. In the case of PCLé fibers containing CNC, CNC was finally added at 1%wt based on PCL weight. Solutions were gently stirred until homogenous solution was obtained. Parameters used were: distance needle-collector 12 cm, voltage 6–7 kV, flow rate 0.4 mL h^−1^, collector speed 500 rpm, capillary speed 0.15 m s^−1^, and application time of 2.5 h. The resulting bilayers PLA/PCL, PLA/PCL-Q, and PLA/PCL-Q-CNC films were named “B”, “B-Q”, and “B-Q-CNC”, respectively. These bilayer systems were also analyzed in order to understand some physical properties. 

The last stage focused in the incorporation of the third layer, the bilayer PCLé-coated PLA films were hot-pressed with a neat PLA’ film (25 µm) at 90 °C without pressure for 1 min using a 4122-model press Carver, Inc. (Wabash, IN, USA). Final trilayer PLA/PCL/PLA’, PLA/PCL-Q/PLA’, and PLA/PCL-Q-CNC/PLA’ films were named as “T”, “T-Q”, and “T-Q-CNC”, respectively. 

### 2.3. Characterization of Physical Properties

#### 2.3.1. Optical Parameter of Trilayer Systems

The color properties of the films were measured in the CIELAB space in a Minolta colorimeter CR-410 Chroma Meter (Minolta Series, Tokyo, Japan). The colorimeter was calibrated with a white standard color plate, D65 illuminant and 2° observer (L* = 97.76, a* = −0.04, b* = 1.90). Color parameters were the average from six measurements along each film. The color difference (ΔE*) of each sample was calculated with respect to the PLA film by Equation (1):

(1) $$∆E=\sqrt{{\left(∆a^{*}\right)}^{2}+{\left(∆b^{*}\right)}^{2}+{\left(∆L^{*}\right)}^{2}},$$

where a*, b*, and *L* are the color coordinates, i.e., *L* (lightness), a* (red-green), and b* (yellow-blue).

The opacity values of developed systems were also analyzed. Films were cut into 1 cm × 4 cm pieces, and six replicates of each sample were measured. The absorbance values of each film were obtained with a UV-Vis mini 1240 spectrophotometer (Spectroquant^®^ Pharo 300, Darmstadt, Germany) at a wavelength of 600 nm. The opacity was determined from Equation (2) [34]:

(2) $$\mathrm{Opacity}=\frac{Abs_{600\mathrm{nm}}}{X},$$

where $Abs_{600\mathrm{nm}}$ is the absorbance of films at 600 nm and *X* is the thickness of every film.

#### 2.3.2. Microstructural Analysis of Trilayer Systems

The morphology of bi- and trilayer structures were analyzed using a scanning electron microscope (SEM) JSM-5410 JEOL5 with an accelerating voltage at 10 kV. The surface microstructure of the cryo-fractured cross-sections of bi- and trilayer systems were previously sputtered with a gold layer to make them conductive before observation.

#### 2.3.3. Thermal Properties of Bi- and Trilayer Systems

Differential scanning calorimetry analysis was carried out in a Differential Scanning Calorimeter (Mettler-Toledo model STAR 822e, Greifensee, Switzerland) coupled to a cooling unit (HAAKE EK 90/mt, Newington, CO, USA). Samples were weighed into aluminum capsules using an analytical balance (Mettler Toledo GA135, Greifensee, Switzerland) with a precision of 0.1 mg. The weight of each sample was approximately 6–8 mg. The samples were subjected to a heating process from 0 to 250 °C at a constant speed of 10 °C min^−1^ under nitrogen atmosphere.

The parameters reported were the glass transition temperature (*T_g_*), melting temperature (*T_m_*), cold crystallization temperature (*T_cc_*), enthalpies of melting (Δ*H_m_*) and crystallization (Δ*H_cc_)*, and the percentage of the crystalline fraction of the PCL fiber and films according to each particular case. The crystallinity was calculated from Equation (3) [35] relating the value of the melting heat (Δ*H_m_*) of each analyzed sample and the theoretical heat to melt the polymer with 100% crystallinity, in PLA (Δ*H*_100_ = 93.6 J g^−1^) and in the PCL (Δ*H*_100_ = 139.3 J g^−1^) [35,36].

(3) $$\;X_{c}=\frac{∆H_{m-}\;∆H_{cc}}{∆H_{100\;}\times X_{Polímero}}*100\;\left(\%\right),$$

where *X_c_* is crystalline fraction (%), Δ*H_m_*: enthalpy of melting of the sample (J g^−1^), Δ*H_cc_* is enthalpy of cold crystallization (J g^−1^), and Δ*H*_100_ is melting enthalpy of fully crystalline polymer (J g^−1^).

Thermogravimetric measurements were carried out in a Mettler Toledo Gas Controller GC20 Stare System TGA/DCS thermal analyzer (Schwerzenbach, Switzerland). The experiments were conducted under dynamic mode and under nitrogen atmosphere (flow rate of 50 mL min^−1^). Film samples were heated from room temperature to 600 °C at 10 °C min^−1^. Onset decomposition temperature (*T_onset_*) at 2.5 wt% of mass loss and temperature at the maximum degradation rate (*T_d,max_*) were reported. 

#### 2.3.4. Water Vapor Permeability (WVP) Analysis

WVP values were gravimetrically analyzed at 50% and 90% RH (relative humidity) and 23 °C using aluminum permeability cups in accordance with the standard method ISO 2528 [37]. The aluminum cups were filled with 7 g of silica gel and sealed with vacuum silicone grease and the films to be tested. The film was fixed in place with a flat Viton ring, an aluminum ring, and three press-screws. Then, relative humidity was ensured by storing the cups in desiccators containing salt solutions: magnesium nitrate (Mg(NO_3_)_2_) and potassium sulfate (K_2_SO_4_) for 50% and 90% RH, respectively. The cups were daily weighed for 2 weeks, and the plot of the weight increment versus time provided the water vapor transmission rate. These values were then divided by the water pressure gradient and film area and multiplied by the sample thickness to obtain the WVP value.

### 2.4. Specific Migration Studies

Release studies of quercetin from bi- and trilayer films were conducted by immersion of the films into two fatty food simulants according to EU Regulation N° 10/2011 about materials and plastic objects in contact with food at 40 °C [38,39]. Double-sided, total immersion migration tests were carried out by total immersion of 3 cm^2^ pieces of each film in 5 mL of food simulant (area-to-volume ratio = 6 dm^2^/L) contained in a glass vial. 

Released Q was analyzed by UV spectroscopy at 370 nm using a Pharo 300 Spectroquant^®^ UV-VIS spectrophotometer (Darmstadt, Germany). The results were expressed as released quercetin concentration into the simulants using an absorbance/concentration (g mL^−1^) calibration curve.

### 2.5. Statistical Analysis

Data were analyzed through an analysis of variance (ANOVA) and Fisher’s multiple range test using the Statgraphics Plus 5.1 program. The experimental design was random type where a *p*-value less than 0.05 indicated significant differences in the measurements between samples and was highlighted with different superscript letters.

## 3. Results

### 3.1. Optical Properties 

**Figure 1:**
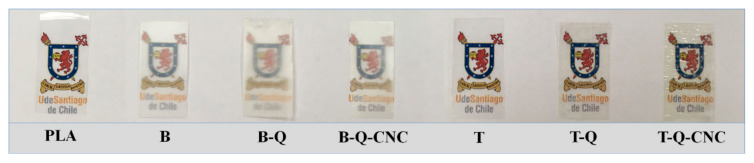
Photographs of developed polymeric systems.

Table 1 contains color parameters and opacity values of bi- and trilayer systems and PLA film. The visual appearance of these structures can be seen in Figure 1 that presents photographs of every polymeric system. 

The collection of electrospun PCL fibers on the PLA’s surface caused a significant decrease in lightness (L*), a slight reduction in a* parameter, and an increase in b* parameter, obtaining a color difference ΔE* = 0.82. Because the trilayer films were obtained through thermal-compression, PCLé melted and the parameters L*, a*, and b* were similar to the values of control PLA, increasing L* and decreasing the color difference ΔE*. The incorporation of Q in the PCLé fibers (samples B-Q and B-Q-CNC) increased b* value due to the yellow tone of this antioxidant agent and resulted in high values of ΔE*. Assuming that the limit value where the human eye perceives the change is ΔE* = 1, a slight yellowish color was observed in these films [40,41]. As Figure 1 shows, this yellowish effect was higher when the fibers were hot pressed and covered by the third PLA’ layer (samples T-Q and T-Q-CNC). These trilayer systems presented higher values of luminosity L* and b* parameter. Yellowish color increased probably due to the release of Q that was encapsulated into the electrospun PCL fibers when the system was hot pressed. The presence of CNC did not alter the color parameter of the materials probably due to its low concentration.

Regarding the bilayer structures, as shown in Figure 1, the electrospun PCL fibers onto the PLA significantly increased the opacity of the bilayer films. The high standard deviation values can be attributed to the fact that the light beam of the spectrophotometer passes through areas with variant presence of PCLé fibers since they were distributed as entangled threads on the PLA. Nonetheless, the trilayer films presented greater transparency because of the melting of the PCLé fibers associated with the hot pressing. The presence of CNC did not affect the transparency of the material.

In general terms, bilayer mats were more opaque than trilayer systems due to the presence of PCLé fibers with their original fibrillar morphologies. However, at the same time, the bilayer mats with Q presented lower color difference than trilayer films because the thermal process caused the melting of PCLé-Q, and therefore, Q was released, increasing the b* parameter.

### 3.2. Morphological Analysis of Developed Trilayer Films

**Figure 2:**
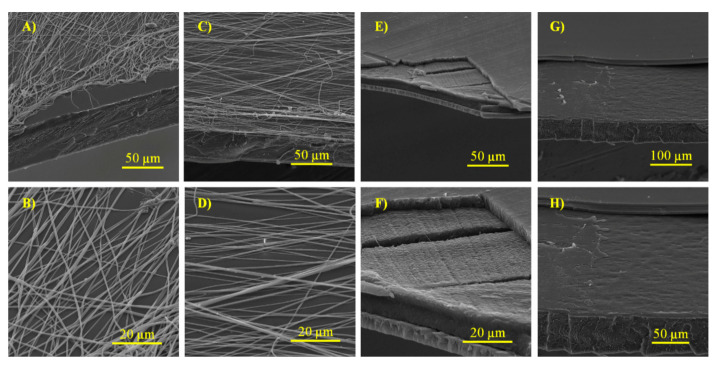
SEM micrographs of developed bi- and trilayer systems containing quercetin (Q) and cellulose nanocrystals (CNC): (**A**,**B**) B-Q; (**C**,**D**) B-Q-CNC; (**E**,**F**) T-Q; and (**G**,**H**) T-Q-CNC.

Figure 2 shows the cross-sections of the developed active bi and trilayers systems at two magnifications. Images A–D of bilayer structures revealed the deposition of the electrospun fibers (PCLé) on the extruded PLA film. Average fiber diameters of PCLé-Q (Figure 2A,B) and PCLé-Q-CNC (Figure 2C,D) were analyzed with image analyzer software (Image J v1.37) and revealed diameters of 689.4 ± 144.2 and 614.8 ± 110.7 nm, respectively. PCLé-Q contained a final composition of 95.24 and 4.76%wt of PCL and Q, respectively, and PCLé-Q-CNC resulted on 94.34, 4.72, and 0.94%wt of PCL, Q and CNC, respectively. The incorporation of the antioxidant and the nanoreinforcements by electrospinning into PCL allowed an easy dispersion on the material on the PLA substrate and certain protection of these compounds. In addition, as Figure 2B,D shows, the deposition with the rotary collector led to an ordering and crossed orientation of the fibers. 

Figure 2A reflects the low adhesion of PCLé-Q fibers on the PLA substrate, thus confirming the need for a third layer application and/or a heat treatment.

Figure 2E–H shows the images of the active trilayer films and the two main layers of PLA that got separated during the cryo-fracture process were clearly observed. The homogenous structure of these PLA layers was observed with greater definition because of their higher thickness. In addition, the loss of the fibrillary morphology of PCLé containing Q and CNC was also confirmed due to the heat treatment caused by the thermal-compression process. Although the thermal treatment applied during the thermo-compression occurred for only one min at 90 °C without pressure, it was sufficient to melt PCL polymer due to its low melting temperature (approximately 60 °C, see Table 2). Therefore, PCLé layer displayed the effect as an adhesive role between the layers.

### 3.3. Thermal Properties

Table 2 shows the main thermal properties of developed films. DSC parameters and crystallinities of bilayer systems (B) and PLA monolayer presented similar values, displaying a *T_g_* around 63 °C and a melting transition with two shoulders, evidencing crystals with different morphologies or lamellar thicknesses [29]. The effect of the incorporation of Q and CNC into PCLé on the thermal properties of the bilayer films was not significant. On the contrary, thermal parameters and crystallinity degrees of trilayer films exhibited significant differences compared to bilayer systems. During the thermal-compression, the heat transference to the films caused a polymer rearrangement evidenced by low Δ*H_cc_* values and highest *X_c_* degrees. Thermal parameters of trilayer systems were similar regardless of the presence of Q and CNC. Nonetheless, the crystallinity diminished when Q and CNC were incorporated in the inner layer, possibly because of steric hindrance and interactions between the components that hindered the chain mobility and reordering during the compression and the cooling process. These interactions could be hydrogen-bond type between hydroxyl, carbonyl groups, and oxygen (ether group) of Q and hydroxyl and ether groups of CNC, or intermolecular interactions between their hydroxyl and carbonyl groups of the polyesters. PCL-Q and PLA-CNC interactions in electrospun active mats have been previously reported [35,42]. Meanwhile, hydrogen-bond type interactions between CNC-Q, PLA-Q, and PCL-CNC have been reported in polymeric films [29,43,44].

Furthermore, *T_g_* values of trilayer films were slightly reduced, attributed to the plasticizing effect of the PCL chains in the inner layer, which owns high flexibility with *T_g_* value of approximately −40 °C [45]. Although both bi- and trilayer films contain PCL, this polymer in the trilayer films suffered a melting process that implied their different behavior as an adhesive between PLA layers, and its plasticizing effect was possibly enhanced. Likewise, the high mobility of PCL chains in the trilayer films caused the cold crystallization and the collapse of the crystals for melting started at slightly lower temperatures than bilayer films and PLA control. The absence of the PCL melting transition in bi- and trilayer mats can be attributed to its low fraction into the films, approximately 2.4% and 1.7% with respect to total weight in the bi- and trilayer films, respectively. Meanwhile, the absence of thermal transitions of CNC and Q is due to their low concentration in the films. Similar results have been observed in zein/PLA bilayer films containing Q and CNC [29].

Conversely, TGA thermograms of all PLA films displayed a single main degradation process regarding the decomposition of PLA chains into carboxylic acids, aldehydes, and lactide monomers and/or oligomers [46]. PCLé also showed a single degradation stage in which a random cleavage of PCL chains produces water, carbon dioxide, and 5-hexenoic, and an unzipping depolymerization produces Ɛ-caprolactone [42]. As Table 3 shows, TGA parameters of PLA film were not affected by the electrospun PCL layer without Q and CNC. However, *T_onset_* was significantly reduced in the trilayer films, possibly due to the fact that flexible PCL chains promoted an earlier network degradation in concordance to DSC analysis. However, the incorporation of Q and CNC reduced the *T_onset_* of bi- and trilayer films due to their decomposition at *T_d.max_* lower than PLA. *T_d_* of Q has been reported around 350 °C attributed to the degradation of the aromatic rings, lower than *T_d,max_* of PLA (364.3 °C) [29,47,48]. Q has also presented a mass loss of 10 wt% approximately up to 160 °C attributed to the elimination of water molecules [47,48]. Despite of lipophilicity of Q, hydroxyl groups of this polyphenolic compound in the solid-state form intermolecular hydrogen bond with water molecules [49]. Q was not degraded by the temperature of hot pressing process because its maximum degradation occurs at 355 °C approx. [29]. Meanwhile, CNC decomposition has been previously reported from 289.6 °C, with maximum degradation rates associated with hydroxyl -CH_2_-OH groups removal at 294 °C, and cellulose chain degradation at 354 °C [29]. In addition, the lower crystallinity degree of trilayer films with Q and Q/CNC promoted their decomposition starting at lower temperatures and produced higher mass loss percentages (see Table 3).

### 3.4. Water Vapor Barrier Properties 

**Figure 3:**
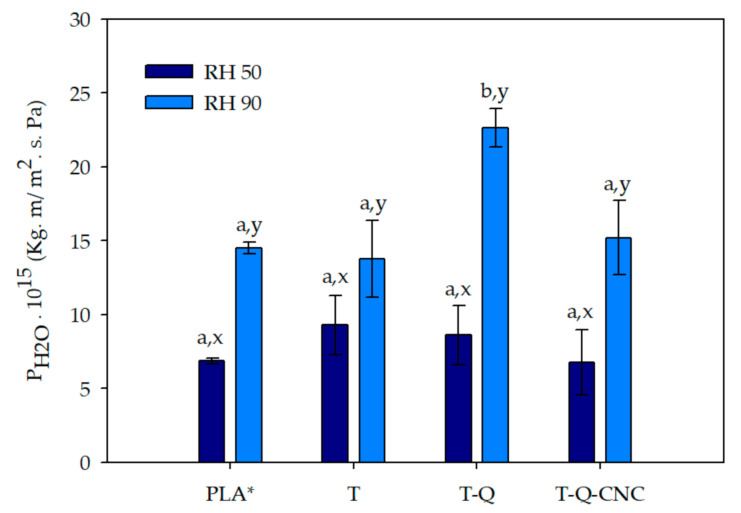
Water vapor permeability values of developed trilayer systems and PLA* (extruded PLA 80 µm). Case letters a,b indicate significant differences among the values of permeability of different films at the same relative humidity (RH); letters x,y indicate significant differences among the values of permeability of the same sample at different values of RH).

In addition to being a vehicle for the incorporation of quercetin for the development of an antioxidant material, the PCLé layer was intended to improve the water barrier property of the resulting material due to the nature of PCL polymer and the incorporation of CNC. Water vapor permeability (P_H2O_) results of the trilayer systems at two relative humidities are shown in Figure 3. At RH 50%, a slight improvement was observed in the films containing CNC, but the differences with other systems were not statistically significant with respect to a control PLA* film, possibly due to the low concentration of this nanoreinforcement with respect to the total volume of the material. P_H2O_ of PLA* film, an 80 µm extruded PLA film, was included in order to have a reference [29]. 

All samples evidenced a considerable increase in permeability values when moving to high relative humidity due to the hydrophilic nature of the polymers. As it was already observed in previous studies, the sorption of water vapor molecules presented a plasticizing effect and the mobility of the polymer chains increased, and, therefore, the diffusivity considerably increased. The incorporation of the thin PCL layer did not have the desired effect, possibly due to its low thickness, which could have been also reduced as a consequence of the thermal-compression process. Interestingly, the incorporation of quercetin dramatically increased at RH 90%. Possibly, the higher interaction between this antioxidant compound and water enhanced its plasticizing effect. The incorporation of CNC counteracted this effect, and T-Q-CNC film exhibited lower permeability values than T-Q at both humidities.

### 3.5. Quercetin Release Studies

**Figure 4:**
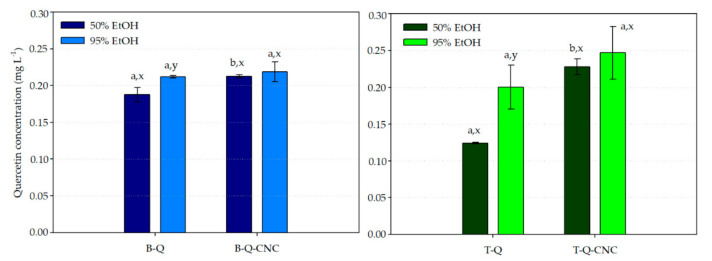
Released Q at the equilibrium condition from B-Q and B-Q-CNC bilayer films (**left**) and T-Q and T-Q-CNC trilayer films (**right**) into EtOH 50% and EtOH 95%. Case letters a,b indicate significant differences among the values of Q released from films of bi- and trilayer systems in the same food simulant, and letters x,y indicate significant differences among the values of Q released from the same film into different food simulants.

Figure 4 presents the results of released quercetin from bi- (B-Q and B-Q-CNC) and trilayer (T-Q and T-Q-CNC) systems in both fatty food simulants at the equilibrium condition for specific migration assays. It is important to mention that, although the thermo-compression process under the described conditions gave rise to a good layer adhesion between films, corresponding layers could have been partially detached when immersed into food simulants during migration tests. A high degree of polymeric swelling was exhibited, promoting and evidencing similar degree of Q release rate between all films. Therefore, significant differences on the diffusion of Q through the films were not observed.

In the case of films without the addition of CNC, the highest values of quercetin concentration at the equilibrium condition in both food simulants were obtained from the bilayer films. This fact could be explained because quercetin in the bilayer films was included into PCLé whose fibrillar and porous structure facilitated the food simulant absorption into their structure, and consequently, the Q release. Conversely, the values of released Q from trilayer T-Q films at the equilibrium condition slightly decreased probably because of the presence of the third PLA’ layer, which could chemically interact with Q and hinder its release. This phenomenon was dependent on the food simulant, obtaining a slightly higher value of Q concentration into EtOH 95% for both B-Q and T-Q films. This fact could be explained in terms of the higher chemical affinity and solubility of this antioxidant into ethanol [30,50]. The lower tendency of quercetin to migrate from the trilayer film could be consequence of several factors associated to the thermodynamic equilibrium for the mass transfer of migrants from polymers. In this case, the main factors could be: (1) lower absorption of food simulant in the PCLé layer due to its third highly crystalline PLA’ layer; (2) a decrease in the quercetin gradient concentration because of the increase in the polymer mass due to the addition of the PLA’ layer; and (3) the availability of some functional groups in PLA for its interaction with quercetin, which increased the affinity of quercetin towards the trilayer film.

Figure 4 also evidenced that the addition of CNC in the bi- and trilayer structures significantly modified the migration tendency of Q released, principally in 95% food simulant, probably because the presence of CNC affected the main structural properties of PCLé, which governed the thermodynamical equilibrium for mass transfer as crystallinity degree (see Section 3.3). Q was released in more extent from the T-Q-CNC trilayer film in both food simulants compared to the values obtained for the T-Q trilayer film. This behavior was dependent on the food simulant. The higher tendency of Q to migrate from the nanoreinforced trilayer films could be related to the fact that the hot-pressing process caused a polymer rearrangement, which could promote specific interactions between the polymers and CNC to the detriment of its interaction with Q. Moreover, the T-Q-CNC trilayer film presented a lower crystallinity degree than the T-Q film, which could promote the sorption of food simulant and the polymer swelling degree and, therefore, the Q release. 

## 4. Conclusions

The combination of different polymeric processing techniques allows the development of biodegradable multilayer systems. Electrospinning was an innovative and simple technique that enabled the homogenous incorporation of quercetin and cellulose nanocrystals, as an active compound and nanoreinforcement, into a thin layer of the biodegradable trilayer film in order to provide antioxidant activity and the improvement of physical properties. The incorporation of a non-volatile antioxidant agent allows the extension of the shelf life and/or effectiveness of this packaging material to long periods, being surely more dependent of the shelf life of both polymers. In this context, this is a weakness on biodegradable packaging that should be addressed.

Results revealed that hot pressing can be an adequate processing technique to assemble polymeric layers, although the stability and adherence between them will be dependent on chemical structure of polymers. Although thermal analysis indicated an increase on crystallinity, trilayer system did not exhibit improvements on water barrier properties. The systems exhibited antioxidant activity in fatty food simulants, and the incorporation of cellulose nanocrystals entailed a higher quercetin release. Developed trilayer systems showed their potential as active materials, but further investigation is needed to be applied as food packaging.

## Figures and Tables

**Table 1:** Chromaticity coordinates, color variation, and opacity index of developed films.

Films	Thickness (µm)	L*	a*	b*	ΔE*	Opacity
PLA	79.4 ± 6.9 ^a^	98.2 ± 0.1 ^d^	−0.05 ± 0.01 ^f^	2.2 ± 0.1 ^a^	–	0.6 ± 0.1 ^a^
B	81.3 ± 6.8 ^a^	97.5 ± 0.1 ^b^	−0.09 ± 0.01 ^e^	2.4 ± 0.1 ^c^	0.8 ± 0.1 ^b^	12.8 ± 1.3 ^d^
B-Q	83.6 ± 8.9 ^a^	97.1 ± 0.1 ^a^	−0.51 ± 0.03 ^c^	3.4 ± 0.1 ^e^	1.7 ± 0.1 ^d^	7.4 ± 2.1 ^c^
B-Q-CNC	83.5 ± 8.1 ^a^	97.2 ± 0.1 ^a^	−0.44 ± 0.02 ^d^	3.2 ± 0.1 ^d^	1.5 ± 0.1 ^c^	7.2 ± 1.9 ^c^
T	99.0 ± 3.5 ^a^	98.3 ± 0.1 ^d^	−0.11 ± 0.01 ^e^	2.3 ± 0.1 ^b^	0.2 ± 0.1 ^a^	3.7 ± 0.3 ^b^
T-Q	102.2 ± 5.2 ^b^	97.9 ± 0.1 ^c^	−0.57 ± 0.02 ^b^	3.9 ± 0.1 ^f^	1.9 ± 0.1 ^e^	3.6 ± 0.6 ^b^
T-Q-CNC	103.7 ± 4.3 ^b^	97.9 ± 0.1 ^c^	−0.61 ± 0.02 ^a^	3.9 ± 0.1 ^f^	1.9 ± 0.1 ^e^	3.1 ± 0.2 ^b^

Lower case letters a–findicate statistically significant differences of the same parameter among films according to ANOVA analysis (*p* < 0.05).

**Table 2:** Differential scanning calorimetry (DSC) parameters of the mats during the first heating process.

Films	*T_g_* (°C)	*T_cc_* (°C)	Δ*H_cc_* (J g^−1^)	*T_m_*_1_ (°C)	*T_m_*_2_ (°C)	Δ*H_m_* (J g^−1^)	*X_c_* (%)
PCLé	–	–	–	59.2 ± 0.8 ^a^	–	108.7 ± 1.1 ^b^	78.0 ± 0.8 ^e^
PLA film	63.5 ± 0.1 ^b^	114.7 ± 0.1 ^bc^	37.3 ± 0.5 ^b^	149.2 ± 0.1 ^c^	153.9 ± 0.3 ^b^	39.2 ± 0.1 ^a^	2.0 ± 0.6 ^ab^
B	63.2 ± 0.5 ^b^	115.1 ± 0.4 ^c^	36.6 ± 0.3 ^b^	149.1 ± 0.4 ^c^	153.6 ± 0.2 ^b^	38.4 ± 0.3 ^a^	1.9 ± 0.1 ^ab^
B-Q	63.1 ± 0.3 ^b^	115.2 ± 0.2 ^c^	33.2 ± 4.1 ^b^	149.3 ± 0.1 ^c^	153.8 ± 0.1 ^b^	36.5 ± 3.9 ^a^	3.6 ± 0.2 ^b^
B-Q-CNC	63.1 ± 0.2 ^b^	114.9 ± 0.1 ^bc^	36.5 ± 1.5 ^b^	148.9 ± 0.2 ^c^	153.6 ± 0.2 ^b^	37.4 ± 1.7 ^a^	1.0 ± 0.2 ^a^
T	61.1 ± 0.1 ^a^	114.5 ± 0.2 ^ab^	20.1 ± 2.1 ^a^	147.4 ± 0.3 ^b^	152.3 ± 0.2 ^a^	38.1 ± 0.6 ^a^	19.1 ± 1.6 ^d^
T-Q	61.1 ± 0.1 ^a^	113.9 ± 0.1 ^a^	23.2 ± 0.2 ^a^	147.1 ± 0.2 ^b^	152.0 ± 0.3 ^a^	38.8 ± 0.1 ^a^	16.7 ± 0.1 ^c^
T-Q-CNC	60.5 ± 0.7 ^a^	114.0 ± 0.5 ^a^	23.7 ± 2.1 ^a^	147.6 ± 0.9 ^b^	151.6 ± 0.7 ^a^	37.4 ± 0.4 ^a^	14.7 ± 1.8 ^c^

Values were reported as the mean and standard deviation of two measurements. Superscripts a–e indicate significant differences through ANOVA analysis (*p* < 0.05).

**Table 3:** TGA parameters of the films.

Films	*T_onset_* (°C)	*T_d,max_* (°C)	Mass Loss at *T_d,max_* (wt%)
PLA film	335.1	364.3	56.4
PCLé fiber	351.4	412.7	56.5
B	335.7	364.3	56.3
B-Q	330.9	363.2	56.5
B-Q-CNC	330.4	366.1	59.2
T	309.5	363.6	59.3
T-Q	304.8	363.7	61.1
T-Q-CNC	305.5	364.3	61.2

## Data Availability

Not applicable.
